# Sterol Regulatory Element-Binding Proteins Are Regulators of the Rat Thyroid Peroxidase Gene in Thyroid Cells

**DOI:** 10.1371/journal.pone.0091265

**Published:** 2014-03-13

**Authors:** Christine Rauer, Robert Ringseis, Susanne Rothe, Gaiping Wen, Klaus Eder

**Affiliations:** Institute of Animal Nutrition and Nutrition Physiology, Justus-Liebig-Universität Gießen, Gießen, Germany; University Claude Bernard Lyon 1, France

## Abstract

Sterol regulatory element-binding proteins (SREBPs)-1c and -2, which were initially discovered as master transcriptional regulators of lipid biosynthesis and uptake, were recently identified as novel transcriptional regulators of the sodium-iodide symporter gene in the thyroid, which is essential for thyroid hormone synthesis. Based on this observation that SREBPs play a role for thyroid hormone synthesis, we hypothesized that another gene involved in thyroid hormone synthesis, the thyroid peroxidase (TPO) gene, is also a target of SREBP-1c and -2. Thyroid epithelial cells treated with 25-hydroxycholesterol, which is known to inhibit SREBP activation, had about 50% decreased mRNA levels of TPO. Similarly, the mRNA level of TPO was reduced by about 50% in response to siRNA mediated knockdown of both, SREBP-1 and SREBP-2. Reporter gene assays revealed that overexpression of active SREBP-1c and -2 causes a strong transcriptional activation of the rat TPO gene, which was localized to an approximately 80 bp region in the intron 1 of the rat TPO gene. *In vitro*- and *in vivo*-binding of both, SREBP-1c and SREBP-2, to this region in the rat TPO gene could be demonstrated using gel-shift assays and chromatin immunoprecipitation. Mutation analysis of the 80 bp region of rat TPO intron 1 revealed two isolated and two overlapping SREBP-binding elements from which one, the overlapping SRE+609/InvSRE+614, was shown to be functional in reporter gene assays. In connection with recent findings that the rat NIS gene is also a SREBP target gene in the thyroid, the present findings suggest that SREBPs may be possible novel targets for pharmacological modulation of thyroid hormone synthesis.

## Introduction

The main function of the thyroid gland is to synthesize the thyroid hormones (TH) thyroxine (T_4_) and triiodothyronine (T_3_). TH synthesis occurs in the thyroid follicles which are the structural units of the thyroid. The thyroid follicles are comprised of a monolayer of follicular epithelial cells (thyrocytes) surrounding the follicular lumen which is filled with colloid. For TH synthesis, iodide is actively taken up across the basolateral membrane of the thyrocyte by the sodium-iodide symporter (NIS) [1]. The iodide is then transported transcellularly and exported through the apical membrane of the thyrocyte. At the apical membrane-colloid interface, thyroid peroxidase (TPO) catalyzes oxidation of iodide by hydrogen peroxide, iodination of tyrosyl residues of thyroglobulin (Tg), a glycoprotein secreted from thyrocytes, and subsequent coupling of the iodotyrosyl residues to form T_3_ and T_4_ [2]. However, TH synthesis is more complex involving not only thyrocytes, but also endothelial cells from adjacent capillaries, and it is well known that TH synthesis is regulated by autocrine/paracrine interactions between endothelial and follicular cells [3]. This complex interplay between thyroid follicles and the microvasculature is best described by the previously developed concept of the angiofollicular units [4], which are considered the morphological-functional units of the thyroid. According to this concept, TH synthesis is the result of a concerted communication between all cellular compartments of the thyroid including those of the microvasculature.

The primary regulator of thyroid growth, differentiation and function is thyrotropin (TSH) which is secreted from a specific subpopulation of pituitary cells, called thyrotropes [5]. The effect of TSH on thyrocytes is mediated via binding to the TSH receptor (TSHR) leading to an increase in intracellular cAMP and stimulation of protein kinase A-mediated pathways. All genes involved in TH synthesis, including NIS, Tg and TPO are activated by TSH thereby stimulating the synthesis and release of TH. Although the TSH/TSHR/cAMP pathway is the most important signaling pathway regulating expression of genes involved in TH synthesis [5]–[7], it was shown that key genes involved in TH synthesis, like NIS and TPO, are also subject to regulation by other signaling pathways, such as the NF-κB pathway [8], [9]. This suggests that TH synthesis is also critically influenced by non-TSH signaling pathways. Noteworthy, the NIS gene was recently reported to be up-regulated by the sterol regulatory element-binding proteins (SREBPs)-1c and -2 [10]. SREBP-1c and SREBP-2 are known as master regulators of fatty acid and triacylglycerol synthesis and cholesterol biosynthesis and uptake, respectively, [11]. Moreover, we have found that TSH causes an up-regulation and activation of SREBPs in thyrocytes, whereas SREBPs are markedly less expressed in thyroid epithelium from TSHR-deficient mice compared to wild-type mice [10]. Our findings provide a plausible explanation for earlier observations that TSH stimulates expression of genes responsible for fatty acid and cholesterol synthesis in thyrocytes [12]–[14], a mechanism aiming to provide membrane lipids for growth and proliferation of thyrocytes which is stimulated by TSH. The SREBPs are synthesized as 120-kDa precursors (pSREBP) located in the endoplasmic reticulum membrane and form a transcriptionally inactive complex with the SREBP cleavage activating protein (SCAP). In the case that the cholesterol content of the cell decreases, the SREBPs are escorted by SCAP to the Golgi, where the SREBPs become proteolytically processed resulting in the release of the transcriptionally active N-terminal domain of the SREBPs (nSREBP). The nSREBPs then translocate into the nucleus where it binds to sterol regulatory element (SRE) binding sites in the regulatory region of target genes, thereby, activating their transcription [10], [15], [16]. Using reporter gene experiments, gel shift assays and chromatin immunoprecipitation we recently identified one functional SRE in the rat NIS gene which is responsible for SREBP-induced activation of NIS gene expression [10]. The importance of the SREBP-dependent regulatory pathway for NIS expression and function in thyrocytes could be evidenced by the finding that inhibition of SREBP maturation results in a reduction of NIS expression and NIS-specific iodide uptake by at least 20% [10]. Based on the observation that SREBPs are novel transcriptional regulators of the NIS gene and therefore play a role for TH synthesis, we hypothesized that another gene involved in TH synthesis, the TPO gene, is also a target of SREBPs. To test this hypothesis we analyzed the TPO 5′-flanking region for putative SREBP binding sites and tested their functionality in reporter gene, gel shift, and chromatin immunoprecipitation experiments. The results show that the rat TPO gene is a SREBP target gene and that SREBP-dependent transactivation is mediated by an approximately 80 bp region within the first intron of the TPO gene which contains two isolated and two overlapping SREBP-binding elements.

## Materials and Methods

### Cell Culture

HepG2 cells (DSMZ, Braunschweig, Germany) and FRTL-5 cells (Cell Lines Service, Eppelheim, Germany) were cultured as described recently [10].

### RNA Isolation and Real-time RT-PCR

For qPCR analysis, FRTL-5 cells were seeded in 24 well plates and incubated as indicated. Total RNA extraction, cDNA synthesis and qPCR were performed as described recently [10]. Gene specific primer pairs and their features are listed in Table 1.

**Table 1:** Characteristics of gene-specific primers used for qPCR analysis.

Gene (NCBI Gene Bank)	Oligonucleotide sequenceForward (5′–3′) Reverse (5′–3′)	PCR product size (bp)
rat SREBP-1c (AF286470.2)	GGAGCCATGGATTGCACATT	191
	AGGAAGGCTTCCAGAGAGGA	
rat SREBP-2 (NM_001033694.1)	CTGACCACAATGCCGGTAAT	204
	CTTGTGCATCTTGGCATCTG	
rat TPO (NM_019353.1)	CAGGTGTTGAGAAGCAGTTG	255
	CTTTGAAAGCTGTAGCCAGG	
rat β-Actin (NM_031144.2)	GACCTCTATGCCAACACAGT	154
	CACCAATCCACACAGAGTAC	

### RNA Interference

For RNAi-mediated gene knockdown of SREBP-1 and SREBP-2, FRTL-5 cells were transiently transfected in 6 well plates with gene-specific Stealth RNAi molecules (“SREBP-1/−2 KO siRNA”) or BLOCK-iT Alexa Fluor Red Fluorescent Control (“Control siRNA”) using LipofectAMINE 2000 (all from Invitrogen) as described recently [10]. 48 h post transfection, total RNA and protein extracts were prepared as described recently [10].

### Protein Extraction and Immunoblotting

Cells were harvested and cytosolic fractions were obtained using the Nuclear Extract Kit from Active Motif (La Hulpe, Belgium). To prevent degradation of SREBPs, FRTL-5 cells were treated with 25 µg/mL of the calpain inhibitor N-acetyl-Leu-Leu-Norleucinal (ALLN) 3 h before cell lysis. Protein concentrations of cytosolic and nuclear extracts were determined by the bicinchoninic acid protein assay kit (Interchim, Montluçon, France) with BSA as standard. 20 µg protein, each from cytosolic and nuclear extracts, were separated by SDS-PAGE (7.5%) and electrotransferred onto nitrocellulose membranes (Pall Corporation, Pensacola, FL, USA). Blotted membranes were incubated with anti-SREBP-1, anti-SREBP-2 and anti-β-Actin (Abcam, Cambridge, UK), followed by a horseradish peroxidase conjugated secondary antibody as reported recently [10]. The signal intensities of specific bands were visualized with ECL Plus (GE Healthcare, München, Germany) and a Bio-Imaging system (Syngene, Cambridge, UK).

### Bioinformatics

For the identification of putative SREBP-binding sites, in silico analysis was performed using the MatInspector software [17] (http://www.genomatix.de/matinspector.html).

### Generation of Plasmids

The 5′-upstream region of the rat TPO gene, using cDNA and genomic DNA sequences from NCBI GenBank (accession no. NM_019353 and AABR06043226), was PCR amplified from rat BAC clone CH230-23A19 (accession no. AC121061, BACPAC Resources, Oakland, USA). The parental reporter gene construct rTPO−1310/+697, corresponding to nucleotide −1310 to +697 relative to the transcription start site, and the 5′-serially deleted reporter gene constructs were generated using different 5′-primers and a common 3′-primer (Table 2), except for the reporter gene construct rTPO+676/+697, which was generated by annealing the oligonucleotides BglII-GATCTCTGGGGTTGCAGTGGGGAAGA and XhoI-TCGATCTTCCCCACTGCAACCCCAGA as described recently [10]. All DNA fragments were subcloned into BglII and XhoI sites of pGL4.10 [luc2] vector (Promega, Mannheim, Germany) upstream of the luciferase reporter gene.

**Table 2:** Oligonucleotides used for PCR amplification of reporter gene constructs from rat TPO.

Oligonucleotide	Oligonucleotide sequence (5′–3′)	PCR product size (bp)
rTPO-BglII_F	TCAAGATCTTCTGGGGTTGCAGTGGGGA	–
rTPO−1310/+697-XhoI_R	ATCCTCGAGGCAAGTGTTAAAGAGGTTAGT	2007
rTPO−1110/+697-XhoI_R	AATCTCGAGGATGTCAATCTGCCTTGGCA	1807
rTPO−719/+697-XhoI_R	AATCTCGAGCGTCTGCTGGGTGAAGTCTC	1416
rTPO+1/+697-XhoI_R	ATTCTCGAGGCACAGCCTGCTTCTTCAGT	697
rTPO+598/+697-XhoI_R	ATTCTCGAGACTTGGGAGGACCCACCTGA	100

Seven additional luciferase reporter vectors, containing two copies of human LDLR-SRE (positive control) [10] or two copies of wild-type and mutated rat TPO 2xSRE+640 or rat TPO 2xInvSRE-like+654 or rat TPO 2xSRE/InvSRE, were generated by annealing the oligonucleotides (Table 3).

**Table 3:** Oligonucleotides used for Annealing of reporter gene constructs from rat TPO.

Oligonucleotide	Oligonucleotide sequence (5′–3′)
rTPO 2xSRE+640-HindIII_F	AGCTCAGCAGAATACTGTGGGATGTACCATAAAATACTGTGGGATGTACCATAAATACCC
rTPO 2xSRE+640-XhoI_R	TCGAGGGTATTTATGGTACATCCCACAGTATTTTATGGTACATCCCACAGTATTCTGCTG
rTPO 2xSRE+640mut-HindIII_F	AGCTCAGCAGAATACTGTGTTACTTACCATAAAATACTGTGTTACTTACCATAAATACCC
rTPO 2xSRE+640mut-XhoI_R	TCGAGGGTATTTATGGTAAGTAACACAGTATTTTATGGTAAGTAACACAGTATTCTGCTG
rTPO 2xInvSRE-like+654-HindIII_F	AGCTGAGATAATCAGAAAGTCAGCAGAATACTGATCAGAAAGTCAGCAGAATACTGTGGGAT
rTPO 2xInvSRE-like+654-XhoI_R	TCGAATCCCACAGTATTCTGCTGACTTTCTGATCAGTATTCTGCTGACTTTCTGATTATCTC
rTPO 2xInvSRE-like+654mut-HindIII_F	AGCTGAGATAATCAGAAAGTCGTATTAATACTGATCAGAAAGTCGTATTAATACTGTGGGAT
rTPO 2xInvSRE-like+654mut-XhoI_R	TCGAATCCCACAGTATTAATACGACTTTCTGATCAGTATTAATACGACTTTCTGATTATCTC
rTPO 2xSRE/InvSRE-HindIII_F	AGCTAAATACCCAGGCCTCTCCTCAGGTGGGTCCCAGGCCTCTCCTCAGGTGGGTCCTCCCA
rTPO 2xSRE/InvSRE-XhoI_R	TCGATGGGAGGACCCACCTGAGGAGAGGCCTGGGACCCACCTGAGGAGAGGCCTGGGTATTT
rTPO 2xSRE/InvSREmut-HindIII_F	AGCTAAATACCCAGGCCTCTCAGATAGTGGGTCCCAGGCCTCTCAGATAGTGGGTCCTCCCA
rTPO 2xSRE/InvSREmut-XhoI_R	TCGATGGGAGGACCCACTATCTGAGAGGCCTGGGACCCACTATCTGAGAGGCCTGGGTATTT

Mutated nucleotides are underlined.

Generation of rat nuclear SREBP-1c (amino acids 1–448) and rat nuclear SREBP-2 (amino acids 1–460) expression plasmids has been described recently [10].

### Transient Transfection

Transient transfections of HepG2 cells were performed as described recently [10] with minor modifications. In brief, cells were seeded in 96 well plates and transiently transfected with 50 ng of the generated reporter gene constructs and co-transfected with 50 ng of either rat nuclear SREBP-1c or rat nuclear SREBP-2 expression plasmids or 50 ng of the empty vector (pcDNA3.1) using FuGENE 6 transfection reagent (Roche Diagnostics, Mannheim, Germany) according to the manufacturer’s protocol. For normalization of transfection efficiency, cells were co-transfected with 5 ng of pGL4.74 [luc2] vector (Promega), which encodes for the Renilla luciferase, as an internal control. In addition, cells were transfected with 50 ng of either pGL4.10 [luc2] vector or pGL4.23 [luc2/minP] vector (both from Promega) or the 2x hLDLR-SRE luciferase reporter vector, containing two copies of the SRE-1 from human LDL receptor, as negative and positive controls. 24 h post transfection, cells were harvested and luciferase activities were measured using Beetle-Juice and Renilla-Juice Kits from PJK (Kleinblittersdorf, Germany). Normalized luciferase activities were calculated by dividing the luciferase activity of each construct by that of the corresponding empty vectors, pGL4.10 or pGL4.23 [18]. Results are shown relative to cells transfected with the empty vector pcDNA3.1 which were set to 1.

### Electrophoretic Mobility Shift Assay (EMSA)

EMSA experiments have been described in detail by Wen et al. [19]. In brief, recombinant rat nuclear SREBP-1c and SREBP-2 proteins were *in vitro* translated from the corresponding expression vectors using the TNT T7 Quick Coupled Transcription/Translation System (Promega) according to the manufacturer’s protocol. Annealed oligonucleotides, spanning the 5′-upstream region of the rat TPO gene from nucleotide +598 to +697, were end-labeled with Digoxigenin (DIG) using the DIG Gel Shift Kit, 2^nd^ Generation from Roche. In addition, annealed and DIG-labeled wild-type and mutated human LDLR-SRE oligonucleotides [10] were used as specific and non-specific control. All sequences of synthetic oligonucleotides are listed in Table 4. For competition experiments, recombinant proteins were incubated with DIG-labeled probes and fold excess of unlabeled specific probes (human LDLR-SRE) as indicated in Figure legends.

**Table 4:** Oligonucleotides used for EMSA.

Oligonucleotide	Oligonucleotide sequence (5′–3′)
rTPO I_F	GATCTCTGGGGTTGCAGTGGGGAAGA
rTPO I_R	TCGATCTTCCCCACTGCAACCCCAGA
rTPO II_F	AGCTTTGCAGTGGGGAAGAGATAA
rTPO II_R	TCGATTATCTCTTCCCCACTGCAA
rTPO III_F	AGCTGATAATCAGAAAGTCAGCAGAATACT
rTPO III_R	TCGAAGTATTCTGCTGACTTTCTGATTATC
rTPO IIImut1_F	AGCTGATAATCAGAAAGTCAGCAGTTCTAG
rTPO IIImut1_R	TCGACTAGAACTGCTGACTTTCTGATTATC
rTPO IIImut2_F	AGCTGATAATCAGAAAGTCGTATTAATACT
rTPO IIImut2_R	TCGAAGTATTAATACGACTTTCTGATTATC
rTPO IIImut3_F	AGCTGATAATCAGATTCGAAGCAGAATACT
rTPO IIImut3_R	TCGAAGTATTCTGCTTCGAATCTGATTATC
rTPO IIImut4_F	AGCTGATAAGATTCAAGTCAGCAGAATACT
rTPO IIImut4_R	TCGAAGTATTCTGCTGACTTGAATCTTATC
rTPO IIImut5_F	AGCTTCGTTTCAGAAAGTCAGCAGAATACT
rTPO IIImut5_R	TCGAAGTATTCTGCTGACTTTCTGAAACGA
rTPO IV_F	AGCTATACTGTGGGATGTACCATA
rTPO IV_R	TCGATATGGTACATCCCACAGTAT
rTPO IVmut_F	AGCTATACTGTGTTACTTACCATA
rTPO IVmut_R	TCGATATGGTAAGTAACACAGTAT
rTPO V_F	AGCTGTGGGATGTACCATAAATACCCAGG
rTPO V_R	TCGACCTGGGTATTTATGGTACATCCCAC
rTPO VI_F	AGCTCAGGCCTCTCCTCAGGTGGGTCCTCCCAAGT
rTPO VI_R	TCGAACTTGGGAGGACCCACCTGAGGAGAGGCCTG
rTPO VImut1_F	AGCTCAGGCCTCTCCTCAGGTGGGTCCTCATGGCT
rTPO VImut1_R	TCGAAGCCATGAGGACCCACCTGAGGAGAGGCCTG
rTPO VImut2_F	AGCTCAGGCCTCTCCTCAGGTGGGGTTGACCAAGT
rTPO VImut2_R	TCGAACTTGGTCAACCCCACCTGAGGAGAGGCCTG
rTPO VImut3_F	AGCTCAGGCCTCTCCTCAGACTAATCCTCCCAAGT
rTPO VImut3_R	TCGAACTTGGGAGGATTAGTCTGAGGAGAGGCCTG
rTPO VImut4_F	AGCTCAGGCCTCTCAGATAGTGGGTCCTCCCAAGT
rTPO VImut4_R	TCGAACTTGGGAGGACCCACTATCTGAGAGGCCTG
rTPO VImut5_F	AGCTCAGGCGGAAACTCAGGTGGGTCCTCCCAAGT
rTPO VImut5_R	TCGAACTTGGGAGGACCCACCTGAGTTTCCGCCTG
rTPO VImut6_F	AGCTTGTAACTCTCCTCAGGTGGGTCCTCCCAAGT
rTPO VImut6_R	TCGAACTTGGGAGGACCCACCTGAGGAGAGTTACA

Mutated nucleotides are underlined.

### Chromatin Immunoprecipitation (ChIP) Assay

ChIP was performed using the Magna ChIP G from Millipore (Schwalbach/Taunus, Germany). PCR amplification of eluted and purified DNA has been described recently [10]. The 270 bp fragment corresponding to the 5′-upstream region of the rat TPO gene, which contains the potential SREBP-binding sites, and a 168 bp fragment corresponding to a random DNA fragment of rat genomic DNA (control) were amplified by using the following primer pairs: rTPO-ChIP_F: TCTGGGGTTGCAGTGGGGA, rTPO-ChIP_R: CCTGAATGTTAGCCATTCACT; control-ChIP_F: TGCTTGCATAGCACCAGGAA, control-ChIP_R: GGAGAAAGCAGAGGACATCA.

### Statistical Analysis

Numerical data were analyzed by one-way ANOVA using the Minitab Statistical Software Rel. 13.0 (Minitab, State College, PA, USA). Differences of *P*<0.05 were considered to be significant.

## Results and Discussion

### Expression of the Rat TPO Gene is Regulated by SREBPs in FRTL-5 Cells

**Figure 1:**
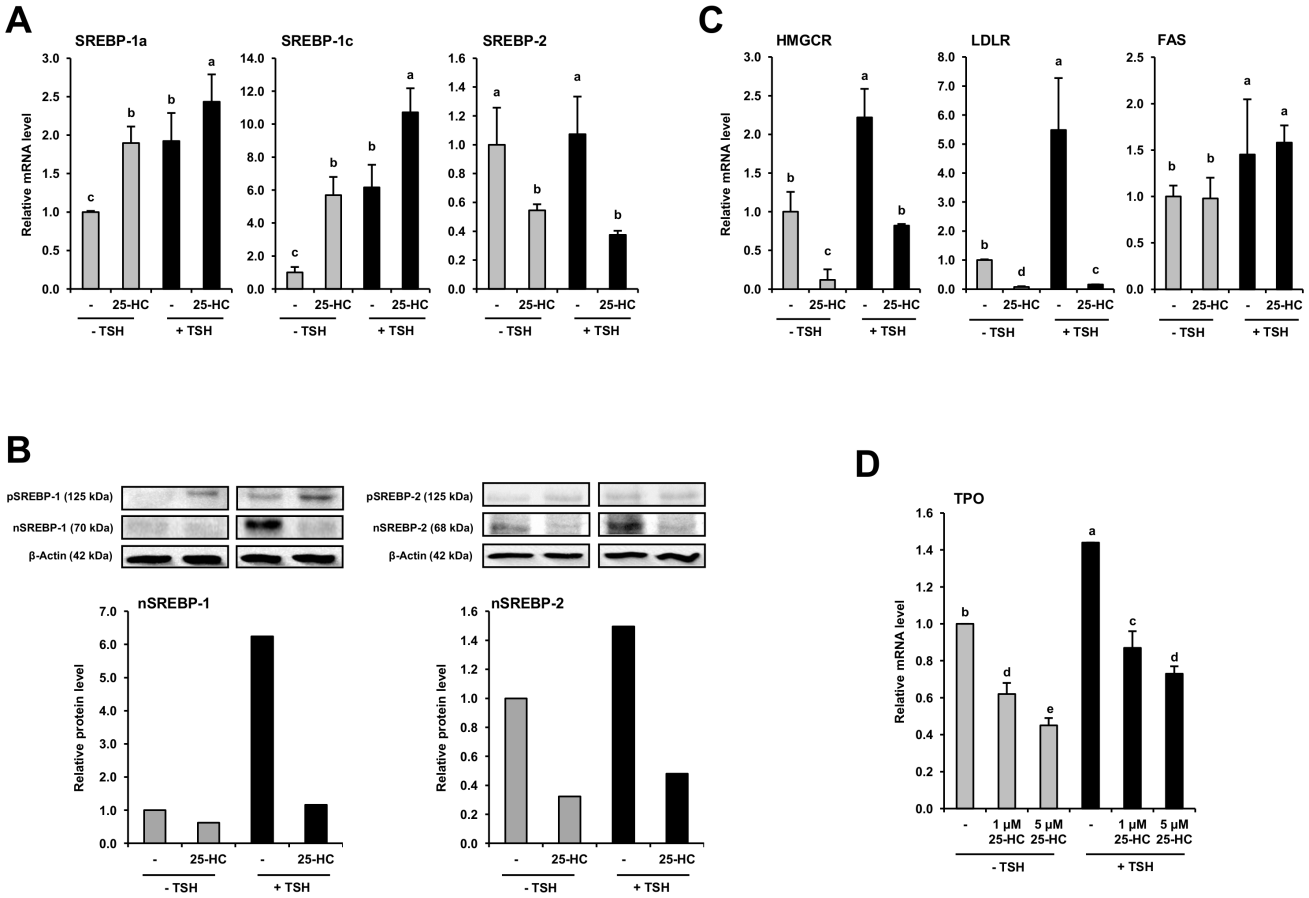
Sterol-mediated inhibition of SREBP maturation reduces expression of rat TPO. FRTL-5 cells were grown in 6H medium until 70–80% confluent, and subsequently treated with 25-HC (1 and/or 5 µmol/L) in the presence and absence of TSH (10 U/L) for 24 h, and analyzed for relative mRNA levels of SREBPs (A), relative protein levels of precursor (p) and nuclear (n) SREBPs (B), and relative mRNA levels of SREBP target genes (HMGCD, LDLR, FAS) (C), and TPO (D). Relative protein levels of pSREBPs and nSREBPs were determined in cytosolic and nuclear extracts, respectively. (B) One representative immunoblot is shown at the top and results from densitometric analysis are given below. (A, C, D) Bars represent means ± SD from three independent experiments and are expressed as fold of control (“−TSH −25HC”). Bars with different lower-case letters differ, P<0.05.

To explore whether SREBPs influence the expression of TPO, FRTL-5 cells were treated with 25-HC (1 and 5 µmol/L), because the activities of all three SREBP isoforms are regulated by cell’s sterol content [20], albeit in a different manner. SREBP-1c, but not SREBP-2, is transcriptionally activated through the activation of liver X receptor by the binding of oxysterols like 25-HC [21]–[23]. However, oxysterols like 25-HC inhibit SREBP-1 and -2 proteolytic processing by blocking the SCAP-mediated movement of SREBPs to the Golgi leading to decreased levels of active nSREBPs [20], [22], [24]. In line with this, we found that the mRNA level of SREBP-1c was elevated by 25-HC, whereas that of SREBP-2 was reduced by 25-HC in FRTL-5 cells (Figure 1A). In addition, we observed that the mRNA level of the SREBP-1a isoform, which is an activator of both, the cholesterol and fatty acid biosynthetic pathway, but is present in much lower amounts in tissues *in vivo* than the other two forms [25], was markedly increased by 25-HC. Using a SREBP-1 antibody, which does not allow to distinguish between the 1a- and 1c-isoform, we demonstrated that the protein level of pSREBP-1 was markedly elevated by 25-HC and that of pSREBP-2 was slightly increased (Figure 1B). In contrast, protein levels of the mature nSREBP-1 and nSREBP-2 were strongly reduced by 25-HC (Figure 1B) indicating that 25-HC inhibits proteolytic processing of SREBP-1 and -2 in FRTL-5 cells, like in McA-RH7777 cells [22]. These experiments were carried out in both, the presence and absence of TSH (10 U/L), because TSH was recently shown to markedly increase mRNA and protein levels of SREBPs in FRTL-5 cells [10]. In agreement with this, mRNA and protein levels of p/nSREBPs were higher in FRTL-5 cells treated with TSH than without (Figure 1A and B). The 25-HC-induced reduction in nSREBP-1 and nSREBP-2 levels was accompanied by a reduction in the mRNA levels of known SREBP-2/−1a target genes (HMGCR, LDLR), but not of the SREBP-1c target gene FAS (Figure 1C) suggesting that 25-HC preferentially inhibits SREBP-2/−1a-dependent gene transcription in FRTL-5 cells. As expected, mRNA levels of TPO were elevated by 24 h treatment with TSH (Figure 1D). However, when FRTL-5 cells were treated with 25-HC, mRNA levels of TPO were dose-dependently reduced by 25-HC both, in the presence and absence of TSH (Figure 1D). This finding provided indication that both, basal and TSH-stimulated expression of TPO is regulated by SREBPs.

**Figure 2:**
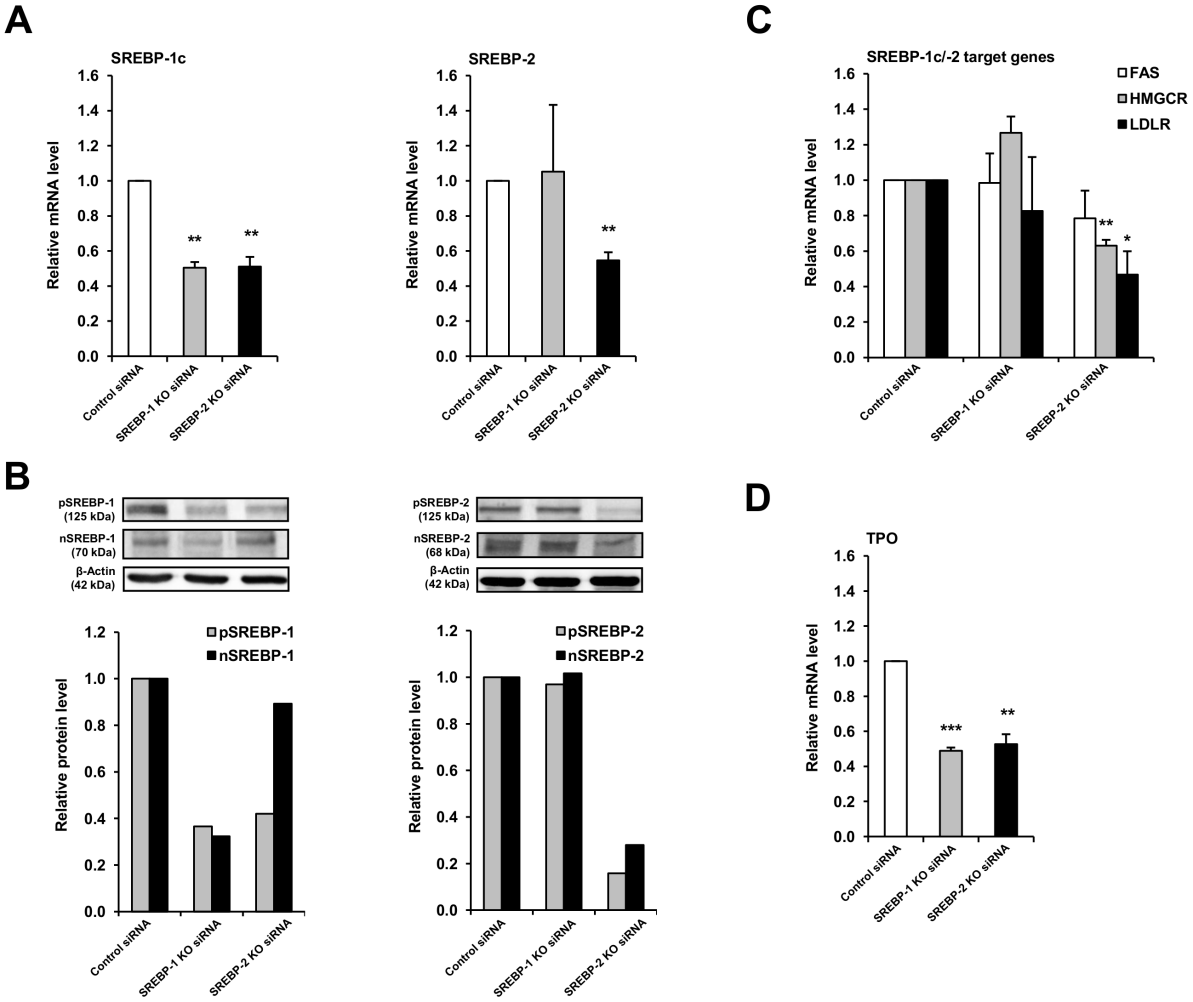
SREBP knockdown reduces expression of rat TPO. FRTL-5 cells were grown in 6H medium until 70–80% confluent, and subsequently transfected with knockdown siRNAs targeting either SREBP-1 or SREBP-2 for 24 h. After transfection, medium was changed to 6H medium for additional 48 h. Relative mRNA levels of SREBP-1c and SREBP-2 (A), relative protein levels of precursor (p) and nuclear (n) SREBPs (B), and relative mRNA levels of SREBP target genes (FAS, HMGCR, LDLR) (C), and TPO (D). Relative protein levels of pSREBPs and nSREBPs were determined in cytosolic and nuclear extracts, respectively. (B) One representative immunoblot is shown at the top and results from densitometric analysis are given below. (A, C, D) Bars represent means ± SD from at least two independent experiments and are expressed as fold of control (“Control siRNA”). *Different from control (“Control siRNA”, P<0.05).

To confirm the importance of SREBPs in regulating TPO expression, we studied the expression of TPO in FRTL-5 cells with a targeted knockdown of either SREBP-1 or SREBP-2. Transfection of FRTL-5 cells with knockdown siRNAs targeting SREBP-1 caused a reduction in the mRNA level of SREBP-1c and protein levels of pSREBP-1 and nSREBP-1 by about 60% after 48 h compared to cells transfected with control siRNAs (Figure 2A and B). Likewise, transfection of FRTL-5 cells with knockdown siRNAs targeting SREBP-2 resulted in a decrease in the mRNA level of SREBP-2 by about 45% and in the protein levels of pSREBP-2 and nSREBP-2 by about 70–80% after 48 h compared to cells transfected with control siRNAs (Figure 2A and B). The siRNA-mediated knockdown of SREBP-2 also resulted in a reduction of SREBP-1c mRNA and pSREBP-1 by about 50% in FRTL-5 cells. This is likely explained by the observation that the SREBP-1c promoter is activated by nSREBP-2 [26]. The mRNA levels of known SREBP-2 target genes (HMGCR, LDLR) were reduced by about 40–50% in FRTL-5 cells transfected with knockdown siRNAs targeting SREBP-2, whereas the knockdown of SREBP-1 did not result in a reduction of the mRNA level of the SREBP-1c target gene FAS (Figure 1C). This again indicated that SREBP-1c-dependent gene transcription may be less important in FRTL-5 cells. However, we have recently observed that the temporal pattern of induction of LDLR and HMGCR by TSH, which causes activation of SREBP-1 and -2, was clearly different from that of SREBP-1c target genes [FAS and glycerolphosphate-acyltransferase (GPAT)]. Namely, the mRNA levels of FAS and GPAT were elevated only at 6 h but not at later time points of TSH treatment, while the mRNA levels of LDLR and HMGCR remained increased from 6 to 24 h of TSH treatment. This indicates that regulation of FAS and GPAT by SREBP-1c in thyrocytes occurs very rapidly and the SREBP-1c-mediated induction of FAS is not observable at later time points. It is therefore possible that the 25-HC-mediated or the SREBP-1 siRNA-mediated inhibition of SREBP-1 maturation led to a reduction of FAS mRNA level at 6 h, but not at 24 or 48 h, which were the incubation periods applied in Figure 1C and 2C, respectively. Future studies are necessary to explain the mechanisms underlying the different response of SREBP-1c and -2 target genes in thyrocytes. Similar as observed in response to inhibition of SREBP maturation by 25-HC, mRNA level of TPO was reduced by about 50% after 48 h, respectively, in response to siRNA mediated knockdown of both, SREBP-1 and SREBP-2 (Figure 2D). These findings again indicated that TPO expression is regulated by SREBPs.

### Nuclear SREBPs Stimulate Transcriptional Activity of the Rat TPO Gene

**Figure 3:**
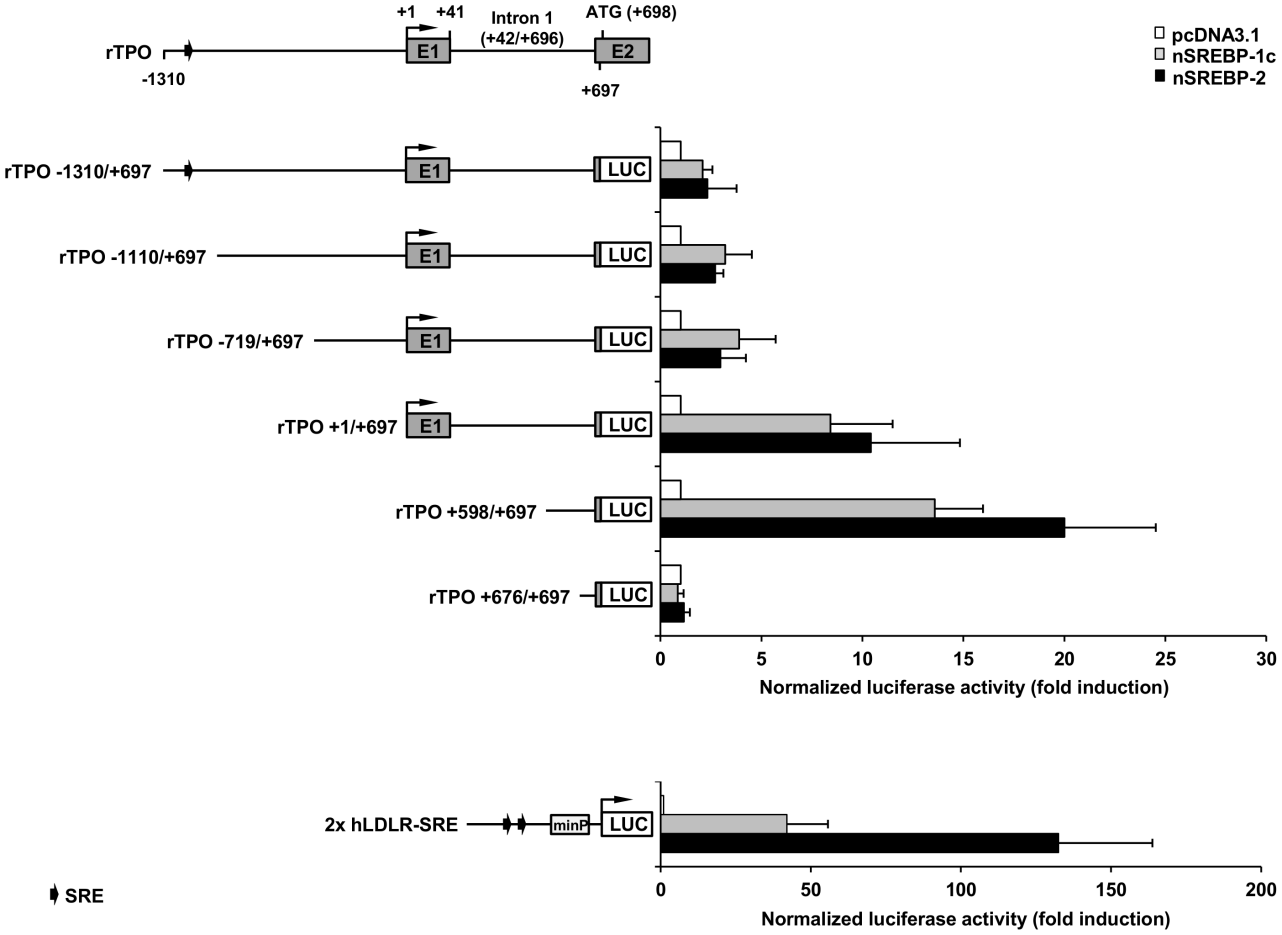
Nuclear SREBP-1c and SREBP-2 stimulate the 5′-flanking region of the rat TPO gene. HepG2 cells were transiently transfected with reporter gene constructs rTPO−1310/+697, rTPO−1110/+697, rTPO−719/+697, rTPO+1/+697, rTPO+598/+697, rTPO+676/+697 or 2x hLDLR-SRE (positive control) and co-transfected with either pcDNA3.1 (empty vector) or plasmids expressing nuclear forms of rat SREBP-1c and rat SREBP-2 for 12 h. After transfection, medium was changed to RPMI1640 medium supplemented with 10% FBS for 24 h. Afterwards, cells were lysed, and luciferase activities were measured. Bars represent means ± SD from at least three independent experiments each performed in quadruplicate. The upper scheme represents the 5′-flanking region of the rat TPO gene from −1310 to +697. Positions of exon 1 (E1), exon 2 (E2) and intron 1 are indicated relative to the transcription start site (+1), which is marked by an arrow. The putative SRE from MatInspector (−1170/−1161) is also indicated.

To examine the SREBP responsiveness of the rat TPO gene we screened an approximately 2000 nt sequence upstream of the translation start site of the TPO gene for the existence of putative SREBP binding sites using MatInspector software (Genomatix). According to this search, one putative SRE (5′-CTCACCTCAC-3′, core similarity = 0.9, matrix similarity = 1.0) could be identified at −1170/−1161 relative to the transcription start site. To evaluate, whether the 2000 bp sequence of the TPO 5′-flanking region is responsive to nSREBPs, we cloned this sequence (from −1310/+697) in front of a firefly luciferase reporter gene, and measured the luciferase activity of this construct rTPO−1310/+697 in response to rat nSREBPs in transient transfection experiments with HepG2 cells. We have recently demonstrated that FRTL-5 cells can be also transiently transfected with SREBP expression plasmids and be successfully used to study activation of SREBP-dependent reporter gene constructs [10]. Despite this and the fact that using a non-thyroidal and non-rat cell model for studying regulation of the rat TPO gene weakens the physiological and the molecular significance of the experimental data, we decided to use HepG2 cells because the reporter response to SREBPs in FRTL-5 cells was clearly less than in HepG2 cells [10], making HepG2 cells a more sensitive model to study SREBP-dependent gene activation, in particular when the response to SREBPs is expected to be lower than for a classical SREBP target gene, like the LDLR. To study activation of the rat TPO gene by SREBPs, we used expression vectors for nSREBP-1c and nSREBP-2, but not nSREBP-1a. Using immunohistochemistry, we have recently shown that SREBP-1 and SREBP-2 are expressed in thyroid follicles of mice [10]. The SREBP-1 antibody used for immunohistochemistry did not enable us to distinguish between the SREBP-1a and -1c isoform, but we assume that the dominant SREBP-1 isoform in the thyroid is SREBP-1c, because it has been reported that SREBP-1c is the predominant SREBP-1 isoform in tissues of animals and humans [25]. In addition, SREBP-1c and SREBP-2 have distinct functions on cholesterogenic and lipogenic genes, respectively, whereas SREBP-1a regulates genes of both pathways. As shown in Figure 3, there was an increase in luciferase activity in response to both, nSREBP-1c and nSREBP-2 in HepG2 cells transfected with rTPO−1310/+697 indicating that the rat TPO 5′-flanking region is responsive to SREBPs, and that this activation is not SREBP isoform-specific. To further find out whether the putative SRE at −1170 or other yet unidentified SREs are responsible for the SREBP responsiveness of the TPO 5′-flanking region, we studied the reporter response to nSREBP-1c and nSREBP-2 of several 5′-deletion TPO constructs, namely rTPO−1110/+697, rTPO−719/+697, rTPO+1/+697, TPO+598/+697, and rTPO+676/+697. We found that the constructs rTPO−1110/+697, rTPO−719/+697, rTPO+1/+697, TPO+598/+697 showed a 3–20 fold increase in reporter response to both, nSREBP-1c and nSREBP-2, whereas construct rTPO+676/+697 was completely unresponsive to SREBPs (Figure 3). These findings indicated that the putative SRE at −1170 is not a functional SREBP binding site and that at least one functional SREBP binding and activation site is present between +598 and +675 of the TPO 5′-flanking region. The finding that the reporter response of the constructs rTPO+1/+697 and TPO+598/+697, which contained only intron 1 sequences downstream of the transcription start site, was markedly greater than that of rTPO−1310/+697, rTPO−1110/+697, and rTPO−719/+697 suggested that the region upstream of the transcription start site of the TPO gene contains binding sites for SREBP co-factors acting as repressors of SREBP transactivation.

### Binding of Nuclear SREBPs to the Rat TPO Gene *in vitro* and *in vivo*

**Figure 4:**
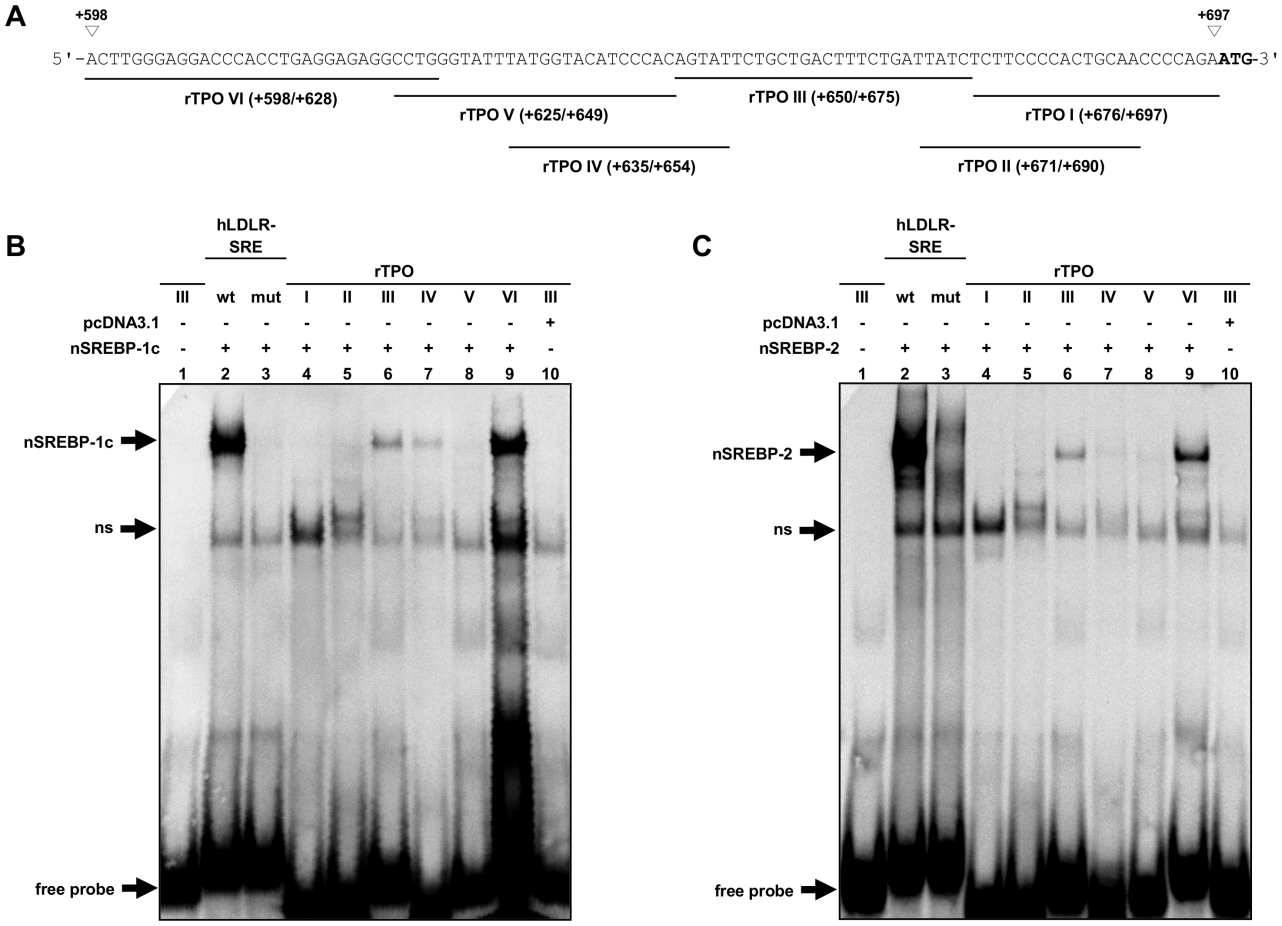
*In vitro*-binding of nuclear SREBP-1c and SREBP-2 to the first intron of the rat TPO gene. (A) Schematic representation of the overlapping oligonucleotide fragments corresponding to the intronic nucleotide sequence of the rat TPO gene between +598 to +697. Positions are indicated relative to the transcription start site. (B, C) *In vitro*-binding of rat nuclear SREBP-1c (B) and rat nuclear SREBP-2 (C) to different sequences of the first intron of the rat TPO gene (rTPO I–VI). EMSA was performed using *in vitro*-translated rat nuclear SREBP-1c or rat nuclear SREBP-2 and DIG-labeled oligonucleotide spanning the intronic nucleotide sequence of the rat TPO gene from +598 to +697. The use of DIG-labeled specific probe (human LDLR-SRE) and non-specific probe (mutated human LDLR-SRE) is also indicated. For illustrating unspecific bands, *in vitro*-translated pcDNA3.1 was incubated with DIG-labeled oligonucleotide corresponding to rTPO III (lane 10).

To further explore whether SREBPs bind to not yet identified binding sites in the SREBP responsive region of the TPO 5′-flanking region, EMSA was performed using labeled double-stranded 20–30 bp oligonucleotides corresponding to different sequences of the TPO 5′-flanking region between +598 and +697 and *in vitro*-translated rat nSREBP-1c and nSREBP-2 (Figure 4A). As shown in Figure 4B and C, a shifted complex could be found between the oligonucleotides rTPO III (+650/+675), and rTPO VI (+598/+628), respectively, and nSREBP-1c and nSREBP-2 (lane 6 and 9, Figure 4B and C). In addition, a shifted complex was observed between rTPO IV (+635/+654) and nSREBP-1c (lane 7, Figure 4B), but not nSREBP-2 (lane 7, Figure 4C). No shifted complex was observed between the other oligonucleotides (rTPO I, rTPO II and rTPO V) and nSREBP-1c and nSREBP-2 (lane 4, 5 and 8, Figure 4B and C). Reliability of the EMSA was tested using oligonucleotides corresponding to the wild-type (positive control) or the mutant human LDLR-SRE (negative control). Using the human LDLR-SRE as specific probe and nSREBPs, we also observed a shifted DNA-protein complex (lane 2 in both, Figure 4B and C), which was not formed between the non-specific probe of the mutant human LDLR-SRE and nSREBPs (lane 3, Figure 4B and C). These results indicated that the first intron of rTPO contains several yet unidentified binding sites for nSREBPs between +598/+628, +635/+654 and +650/+675.

**Figure 5:**
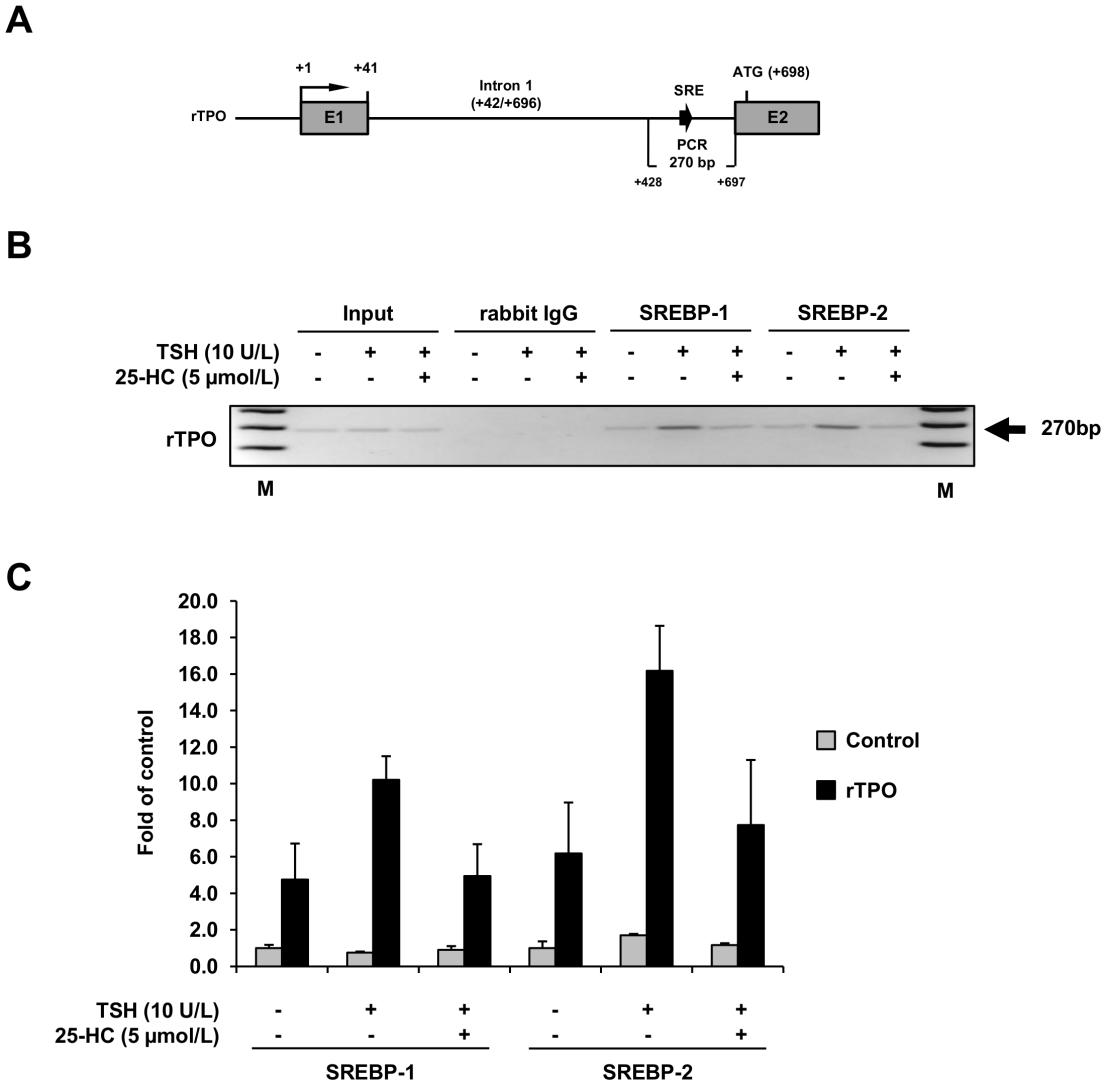
*In vivo*-binding of nuclear SREBP-1c and SREBP-2 to the first intron of the rat TPO gene. Chromatin immunoprecipitation of rat TPO first intron using antibodies against rat SREBP-1 and SREBP-2. A 270 bp fragment from the rat TPO first intron, including the potential SREBP binding sites (A), and a random control sequence were analyzed by conventional PCR (B) and qPCR (C) in the immunoprecipitated chromatin of FRTL-5 cells. FRTL-5 cells were grown in 6H medium in 150 mm dishes until 70–80% confluent, then switched to 5H medium (without TSH) for 5 d, and subsequently treated with 25-HC (5 µmol/L) in the absence or presence of TSH (10 U/L) for 24 h. Rabbit IgG was used as control. The image from agarose gel electrophoresis is representative for one out of three independent ChIP experiments each providing similar results. Data from qPCR analysis represent means ± SD for the three independent experiments. M, DNA fragment size marker.

To clarify whether SREBP-1c and SREBP-2 is bound to the rat TPO intron 1 sequence containing the yet unidentified SREBP binding sites *in vivo*, ChIP was performed using antibodies against rat SREBP-1 and SREBP-2. Chromatin was isolated from FRTL-5 cells treated for 24 h either without TSH, with TSH (10 U/L) alone or with TSH (10 U/L) and 25-HC (5 µmol/L) in parallel. As illustrated in Figure 5, a 270-bp fragment of TPO intron 1 sequence spanning the +598/+675 sequence (Figure 5A) could be amplified from TSH treated cells when immunoprecipitation was performed with antibodies against SREBP-1 and SREBP-2, but not with non-specific rabbit IgG (Figure 5B and C). In contrast, only negligible amplification of the 270-bp fragment was able from cells treated without TSH or co-treated with TSH and 25-HC (Figure 5B and C). As expected, no amplification of a random control sequence (168 bp) occurred when immunoprecipitation was carried out with SREBP-specific antibodies (not shown). These data suggested that SREBP-1c and SREBP-2 binds *in vivo* to the TPO intron 1 sequence +598/+675 containing the yet unidentified SREBP binding elements.

### Identification of SREBP-responsive cis-elements in the Rat TPO Gene

**Figure 6:**
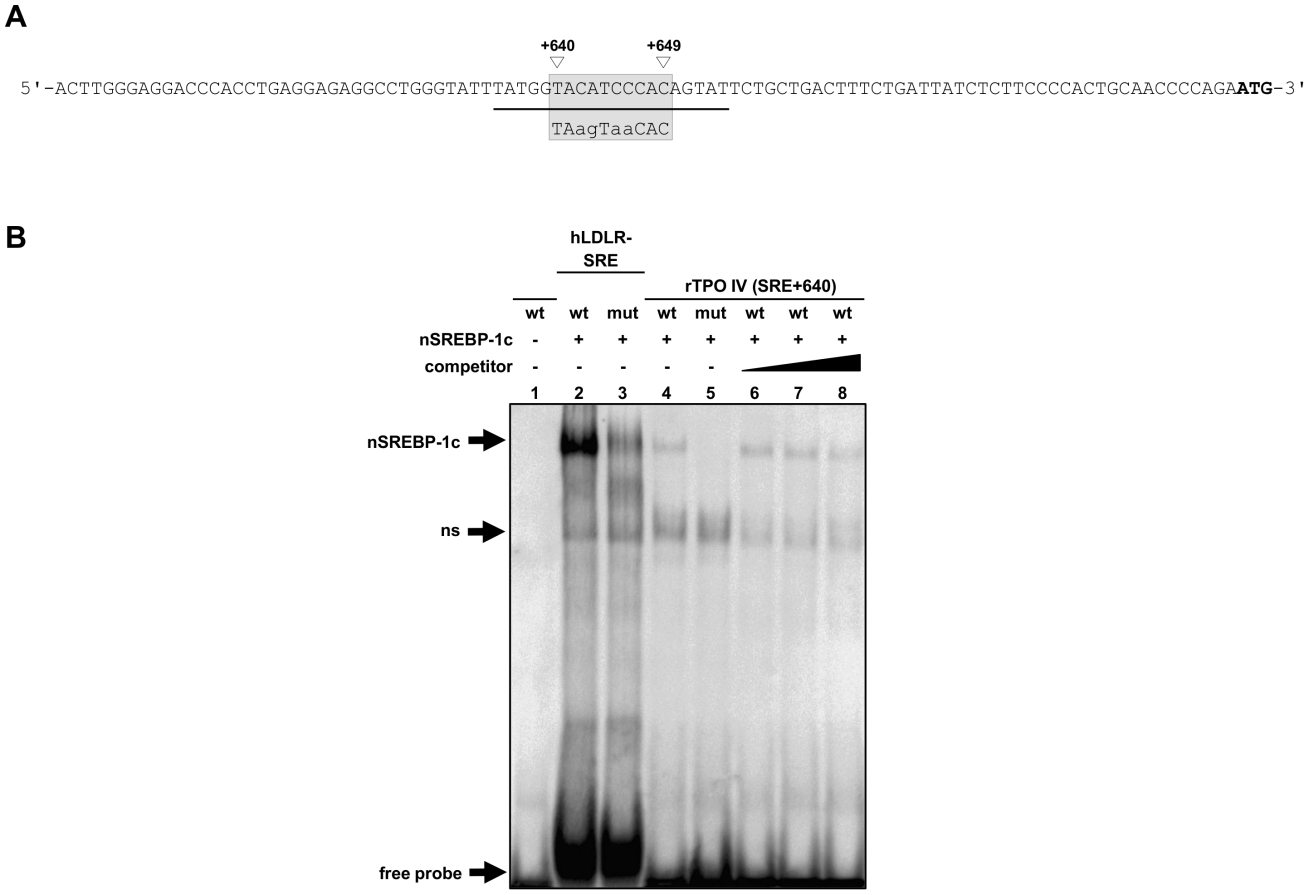
*In vitro*-binding of rat nuclear SREBP-1c to rTPO IV. (A) Nucleotide sequence of rTPO IV, containing the SRE+640, is underlined. The wild-type (upper strand) and mutated (lower strand) sequences of the SRE+640 are in shaded boxes. The lower-case letters represents the substituted nucleotides. (B) *In vitro*-binding of rat nuclear SREBP-1c to the SRE+640 in the rat TPO first intron. EMSA was performed using *in vitro*-translated rat nuclear SREBP-1c and DIG-labeled oligonucleotide corresponding to either wild-type or mutated SRE+640. For competition, 10-, 20- and 50-fold molar excess of unlabeled specific probe (human LDLR-SRE) was used. DIG-labeled specific probe (human LDLR-SRE) and non-specific probe (mutated human LDLR-SRE) is also indicated.

Since sequence alignment with the classic SRE-1 from human LDL receptor (5′-ATCACCCCAC-3′; [27]) revealed one cis-element (5′-TACATCCCAC-3′) with relatively high sequence homology (70%) within rTPO IV (+635/+654) at +640/+649 (Figure 6A), we studied the specificity of SREBP-1c binding to this sequence using EMSA. Figure 6B shows that a shifted complex was formed between nSREBP-1c and the wild-type rTPO IV (+635/+654) (lane 4), but not with a mutant rTPO IV (lane 5), in which four nucleotides within the cis-element at +640/+649 were mutated. Competition experiments revealed that complex formation was successively reduced with increasing molar excess of unlabeled specific probe (wild-type human LDLR-SRE) (lane 6–8). This result indicated that SREBP-1c binds specifically to the cis-element at +640/+649, designated as SRE+640.

**Figure 7:**
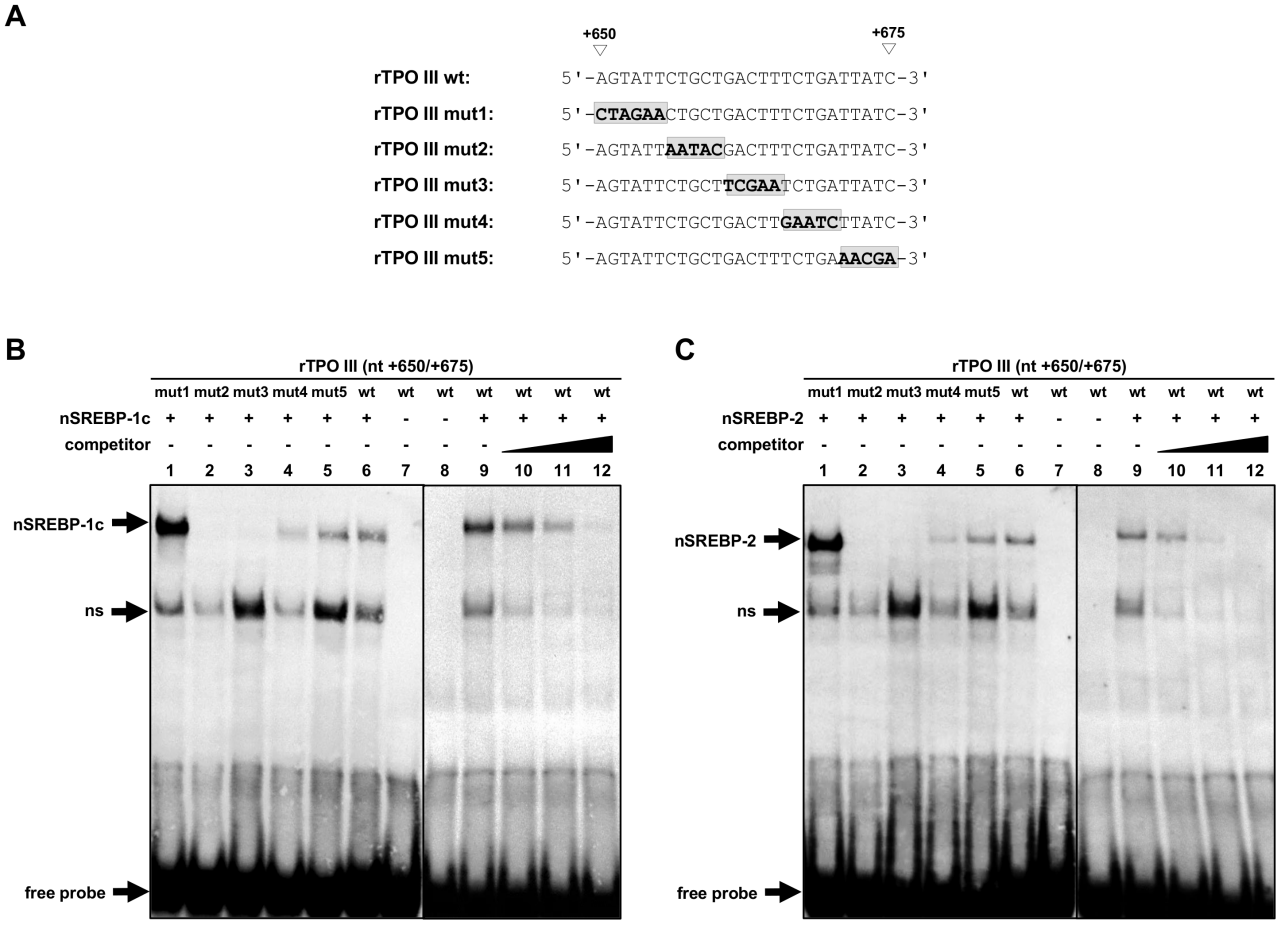
Identification of a SREBP-responsive element within rTPO III (+650/+675) by mutational and competitive analysis. (A) Nucleotide sequences of either wild-type or mutated rTPO III used for EMSA. The mutated nucleotides are highlighted and in shaded boxes. (B, C) *In vitro*-binding of rat nuclear SREBP-1c (B) and rat nuclear SREBP-2 (C) to rTPO III. EMSA was performed using *in vitro*-translated rat nuclear SREBP-1c or rat nuclear SREBP-2 and DIG-labeled oligonucleotide corresponding to rTPO III (+650/+675). For competition, 50-, 100- and 250-fold molar excess (SREBP-1c) or 25-, 50- and 100-fold molar excess (SREBP-2) of unlabeled specific probe (human LDLR-SRE) was used.

To identify the SREBP-responsive cis-element within position +650/+675, also EMSA experiments were performed with a set of oligonucleotides bearing 5 to 6 bp successive mutations and either nSREBP-1c or nSREBP-2 (Figure 7A). Whereas a shifted protein/DNA complex was observed between both nSREBP isoforms and the wild-type rTPO III wt (lane 6) and the mutant rTPO III mut1 (lane 1), rTPO III mut4 (lane 4) and rTPO III mut5 (lane 5), the mutant rTPO III mut2 (lane 2) and rTPO III mut3 (lane 3) lost the ability to form a shifted complex with nSREBP-1c (Figure 7B) and nSREBP-2 (Figure 7C). This indicated that the putative cis-element for both, SREBP-1c and SREBP-2, is located between +656 and +665. To confirm the binding specificity of both SREBP isoforms to the +650/+675 sequence, competition experiments were performed using the wild-type rTPO III wt and increasing molar excess of unlabeled specific probes (wild-type human LDLR-SRE) (Figure 7B and 7C). These experiments revealed that complex formation was successively reduced with increasing molar excess of unlabeled specific probe (lanes 10–12). At the highest molar excess of unlabeled probe (lane 12), complex formation was almost completely absent being indicative of complete competition.

**Figure 8:**
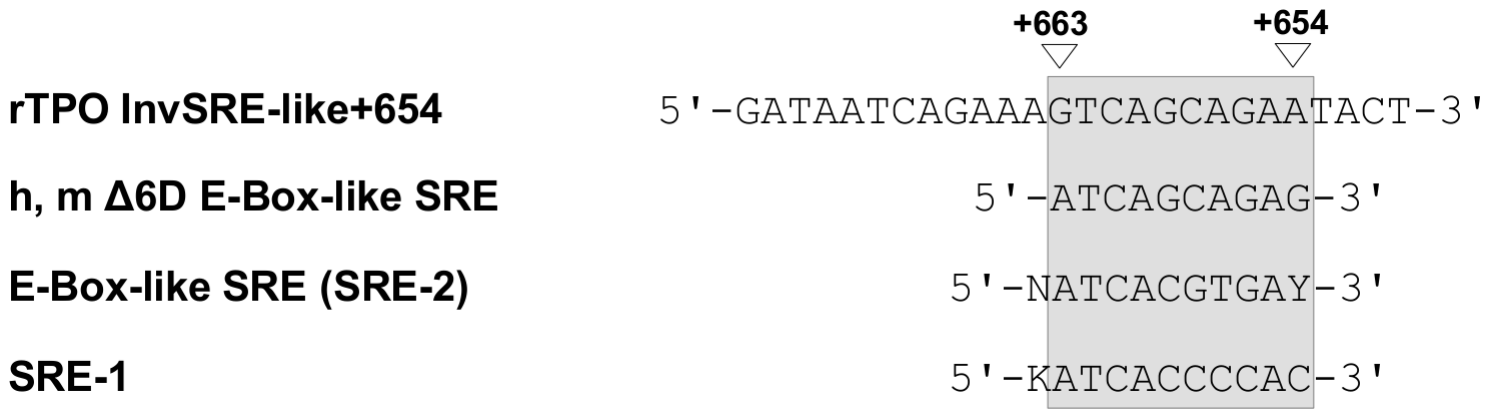
Sequence comparison of the SREBP-responsive sequence within rTPO III. Comparison of the antisense strand of rTPO III with the consensus sequences of classic SRE-1 and E-Box-like SRE (SRE-2) and with the E-Box-like SRE of the h, m Δ6-desaturase gene [21].

Sequence comparison of the sense and antisense strand of the SREBP-responsive sequence between +656 and +665 with identified SREBP binding elements from known SREBP target genes revealed an inversely oriented sequence from +654 to +663 that had 50% sequence homology with the classic SRE-1 from human LDL receptor. In addition, our newly identified SREBP-responsive sequence showed characteristics of both, a SRE and an E-box. Such elements are called SRE-2 or SRE-like and are frequently found in the promoter region of lipogenic genes [26], [27]. Therefore, we designated our identified SRE as InvSRE-like+654. This SRE showed high homology (80%) with a functional E-box-like SRE (SRE-2) identified in the promoter of the lipogenic human and mouse Δ6-desaturase gene [28] (Figure 8).

**Figure 9:**
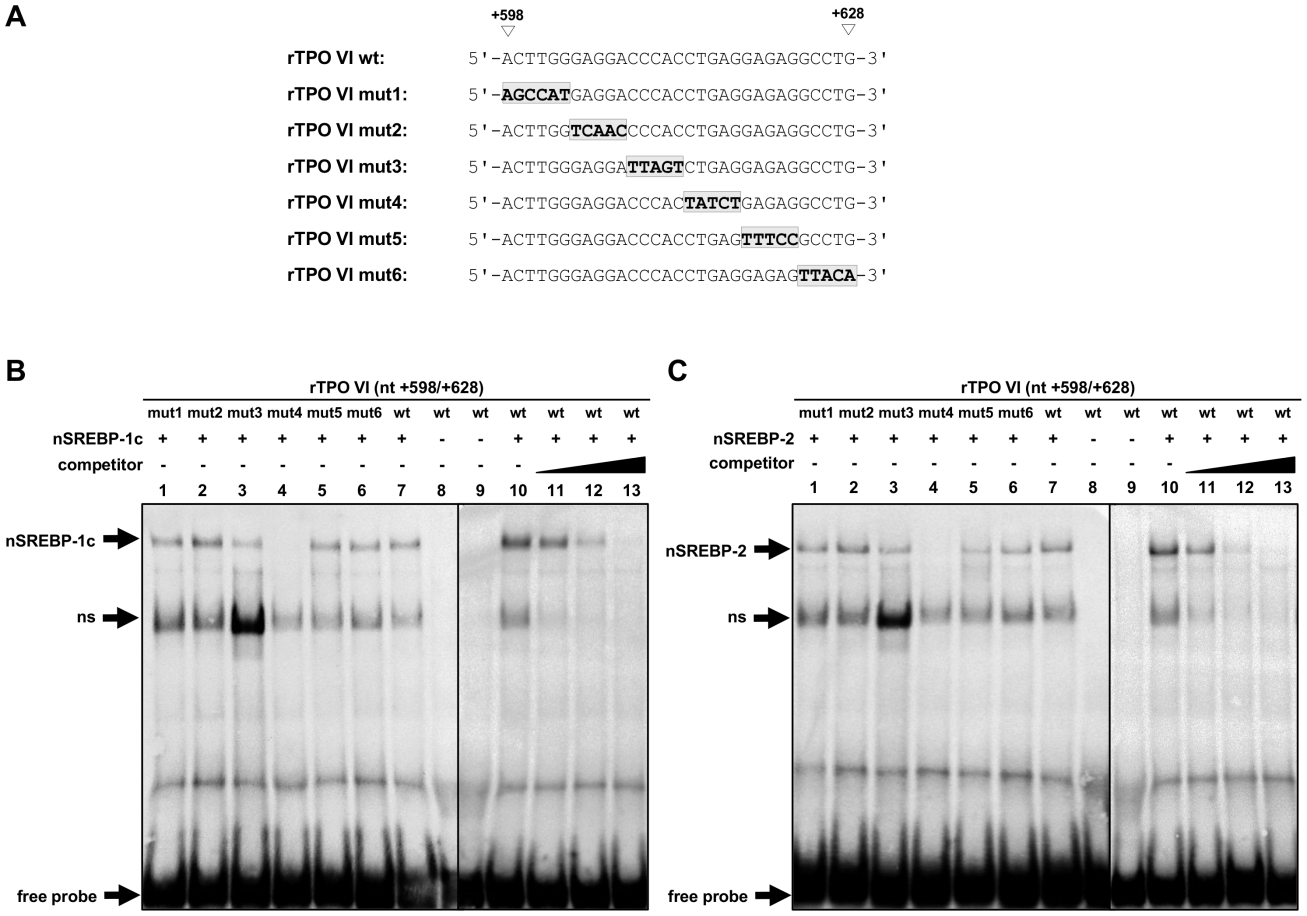
Identification of a SREBP-responsive element within rTPO VI (+598/+628) by mutational and competitive analysis. (A) Nucleotide sequence of either wild-type or mutated rTPO VI used for EMSA. The mutated nucleotides are highlighted and in shaded boxes. (B, C) *In vitro*-binding of rat nuclear SREBP-1c (B) and rat nuclear SREBP-2 (C) to rTPO VI. EMSA was performed using *in vitro*-translated rat nuclear SREBP-1c or rat nuclear SREBP-2 and DIG-labeled oligonucleotide corresponding to rTPO VI (+598/+628). For competition, 50-, 100- and 250-fold molar excess (SREBP-1c) or 25-, 50- and 100-fold molar excess (SREBP-2) of unlabeled specific probe (human LDLR-SRE) was used.

The same approach as for the identification of the SREBP-responsive cis element within +650/+675 was applied to identify a SREBP-responsive cis-element within +598/+628. EMSA revealed that a shifted protein/DNA complex was formed between nSREBP-1c (Figure 9B) and nSREBP-2 (Figure 9C) and the wild-type rTPO VI wt and the mutant rTPO VI mut1 (lane 1), mut2 (lane 2), mut3 (lane 3), mut5 (lane 5), and mut6 (lane 6). In contrast, the mutant rTPO VI mut4 (lane 4) lost the ability to form a shifted complex with nSREBP-1c (Figure 9B) and nSREBP-2 (Figure 9C). This finding suggested that the nucleotides +614 to +618 are critical for the binding of both, nSREBP-1c and nSREBP-2. Again the binding specificity of both SREBP isoforms to the +598/+628 sequence was confirmed in competition experiments with the wild-type rTPO VI oligonucleotide and increasing molar excess of unlabeled specific probes (wild-type human LDLR-SRE). Fig. 9B and 9C shows that formation of the shifted complex successively decreased with increasing molar excess of unlabeled specific probe (lanes 11–13).

**Figure 10:**
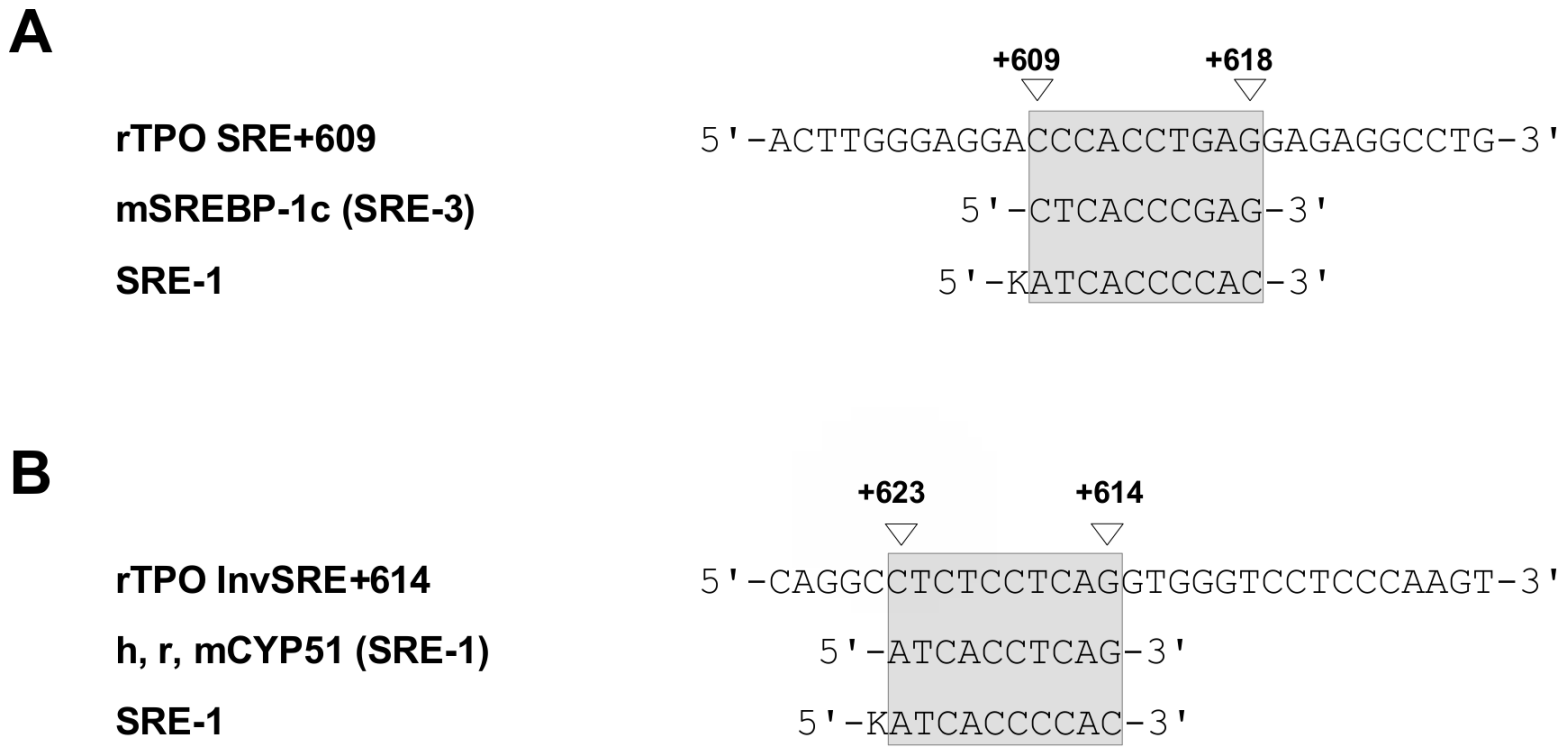
Sequence comparison of the SREBP-responsive sequence within rat TPO VI. (A, B) Comparison of the sense (A) and antisense (B) strand of rTPO VI with the consensus sequence of classic SRE-1 and with SRE-3 of mSREBP-1c [22] and SRE-1 of h, r, mCYP51 [23].

Sequence analysis of the sense and antisense strand of the SREBP-responsive region between +614 and +618 and its adjacent region revealed two overlapping putative SREBP-binding motifs (Figure 10). The sense strand contained a sequence from +609 to +618 which was 50% homologue to the classic SRE-1 from human LDL receptor, but 80% homologue to the SRE-3 of the murine SREBP-1c promoter (Figure 10A) [29]. The second, inversely oriented binding sequence was located at +614/+623 and shared 60% homology with the classic SRE-1 from human LDL receptor and 80% homology with a functional SRE identified in the human, rat and mouse CYP51 promoter (Figure 10B) [30]. We designated these two overlapping SREBP-responsive sequence as InvSRE+614 and SRE+609.

### Sequence Comparison of the SREBP-responsive cis-elements in the TPO Gene between Rat and Mouse

**Figure 11:**
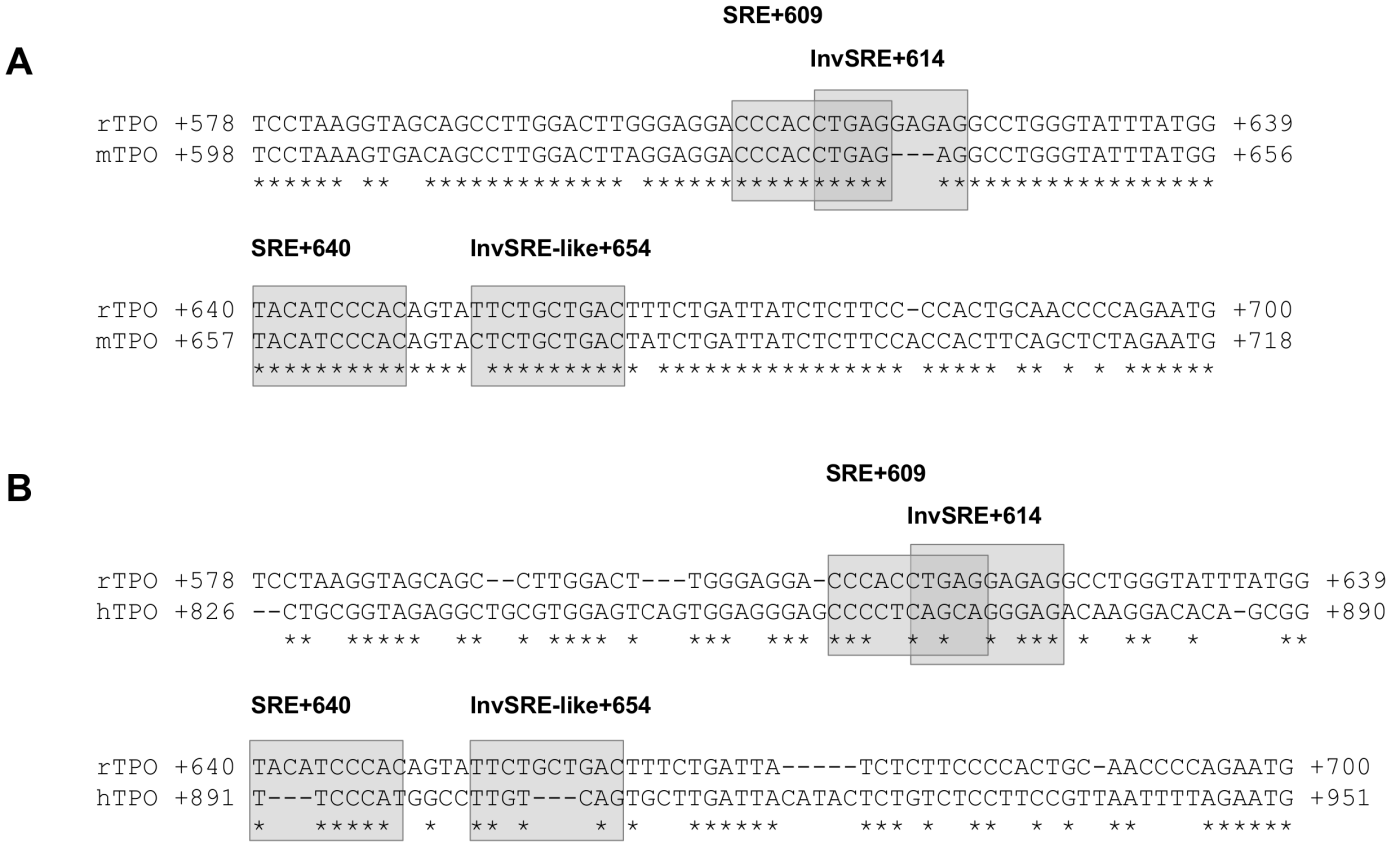
Sequence alignment of the SREBP-responsive cis-elements in the TPO gene between rat and mouse. Alignment of the intronic sequence of rat TPO gene from +578 to +700 (NM_019353) with the intronic sequence of mouse TPO gene from +598 to +718 (NM_009417) (A), and with the intronic sequence of human TPO gene from +826 to +951 (NM_001206745) (B). Conserved nucleotides are indicated by asterisks and the identified SREBP-responsive cis-elements are in shaded boxes.

It is well known that nucleotide sequences, which are critical for gene regulation, typically display a high degree of conservation. Thus, such sites can be successfully predicted from genome comparison of closely related species like rats and mice [31]. To provide further indication of the regulatory importance of the identified SREBP-responsive elements, we carried out sequence alignment of the identified SREBP-responsive regions of rat TPO gene with the mouse TPO gene. As shown in Figure 11A, we observed that the identified SRE+609 and SRE+640 of the rat TPO gene are completely conserved (100% homology) in the mouse TPO gene. The InvSRE+614 and the InvSRE-like+654 were 70% and 90%, respectively, homologue to the mouse TPO gene also indicating a high degree of conservation of the sequence and position between mouse and rat. Thus, these additional findings from sequence comparisons together with our experimental findings substantiate the importance of the identified SREBP-responsive elements for transcriptional regulation of the rat TPO gene. However, sequence alignment of the identified SREBP-responsive regions of rat TPO gene with the human TPO gene showed a markedly lower degree of homology (Figure 11B). This indicates that transcriptional regulation of the human TPO gene by SREBPs may differ from that of the rat and mouse TPO gene. To clarify this future studies are required.

### Evaluation of the Transactivation Activity of the Identified SREBP-responsive cis-elements of the Rat TPO Gene

**Figure 12:**
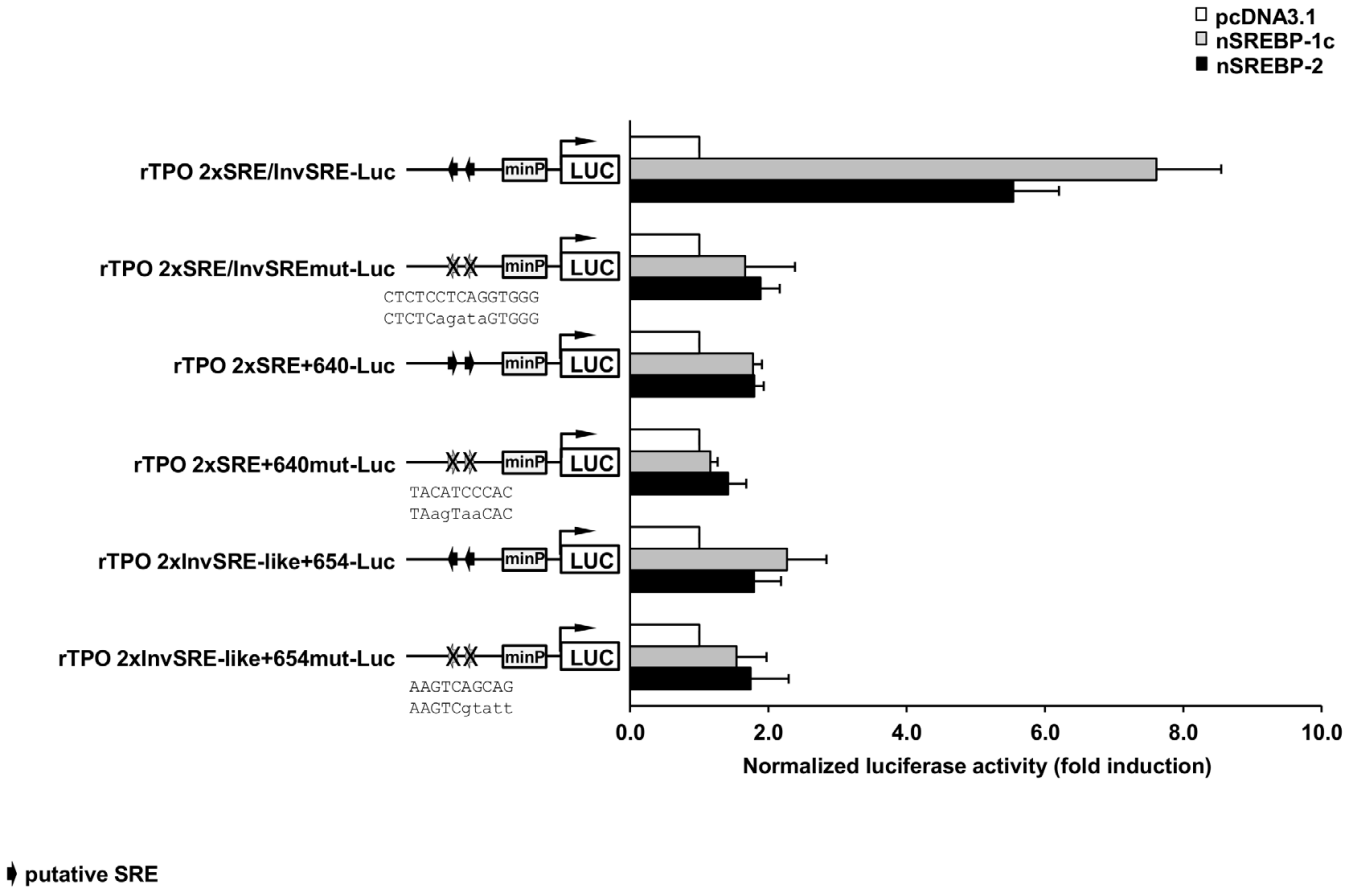
Evaluation of the transactivation activity of the identified SREBP-responsive cis-elements of the rat TPO gene. HepG2 cells were transiently transfected with either wild-type or mutated reporter gene constructs of rTPO 2xSRE/InvSRE-Luc, rTPO 2xSRE+640-Luc, rTPO 2xInvSRE-like+654-Luc and co-transfected with either pcDNA3.1 (empty vector) or plasmids expressing nuclear forms of rat SREBP-1c and rat SREBP-2 for 12 h. After transfection, medium was changed to RPMI1640 medium supplemented with 10% FBS for 24 h. Afterwards, cells were lysed, and luciferase activities were measured. Bars represent means ± SD from at least three independent experiments each performed in quadruplicate. The wild-type (upper strand) and mutated (lower strand) sequences of the putative SREBP-responsive cis-elements are indicated. The lower-case letters represents the substituted nucleotides.

Several studies demonstrated that SREBP-binding elements identified in a given SREBP target gene are not mandatorily functional, despite binding of SREBPs was shown using EMSA [32]–[34]. In order to test whether our identified SREBP-binding elements SRE+640, InvSRE-like+654 and the overlapping SRE+609/InvSRE+614 are capable of mediating SREBP-dependent transactivation of the rat TPO gene, we prepared three luciferase reporter constructs containing two copies of each of these in front of the luciferase reporter. Transient transfection of these constructs and co-transfection of either nSREBP-1c or nSREBP-2 expression plasmids or empty vector (pcDNA3.1) into HepG2 cells showed that only the wild-type construct containing the overlapping SRE+609/InvSRE+614 was responsive to nSREBP-1c and nSREBP-2 (Figure 12), but not the wild-type constructs containing two copies of either SRE+640 or InvSRE-like-654. In addition, the mutant versions of all these constructs, in which five nucleotides had been substituted, did not show any response at all to nSREBP-1c and nSREBP-2. These findings indicated that only the overlapping SRE+609/InvSRE+614 is a functional SREBP binding and activation site, at least in the reporter gene assay. The core motif of the overlapping SRE+609/InvSRE+614, 5′-CTGAG-3′and its inverse sequence 5′-CTCAG-3′, shared 50% each of the SRE+609 and the InvSRE+614, wherefore it was not possible to solely ascribe the SREBP-dependent transactivation ability to one of these two binding elements. However, given that the degree of conservation between rat and mouse was higher for the SRE+609 (100% homology) than for the InvSRE+614 (70% homology), it is likely that the SRE+609 is responsible for SREBP-dependent transactivation of the rat TPO gene. The other binding sequences, SRE+640 and InvSRE-like+654, were shown to be not functional in the reporter gene assay indicating that these are SREBP binding sites but not SREBP transactivation sites. Nevertheless, we cannot exclude that SRE+640 and/or InvSRE-like+654 are functional *in vivo*, because we tested the transactivation ability of these bindings elements in isolated form in the reporter gene assay, but it is possible that SRE+640 or InvSRE-like+654 or both of them work in concert with SRE+609/InvSRE+614 and their interaction is necessary to allow the complete SREBP-dependent transactivation potential of the rat TPO gene *in vivo*.

Despite providing evidence for regulation of TPO gene expression by SREBPS, the present study has several limitations: One important limitation regards the physiological and the molecular significance of SREBP-dependent regulation of the TPO gene in the thyroid, because we used a non-thyroidal and non-rat cell model for the reporter gene experiments. In light of the fact that sequence alignment of the identified SREBP-responsive regions between rat and human revealed a low degree of homology and many instances of tissue-specific regulation of gene expression are known from the literature, it is important to investigate the transcriptional regulation of the rat and the human TPO genes by SREBPs in suitable rat and human, respectively, thyrocyte models in future studies. In addition, despite showing that the TPO mRNA level is subject to regulation by SREBPs in the rat FRTL-5 thyrocyte model, the functional significance for TH synthesis cannot be evaluated from this thyrocyte model because the FRTL-5 model is notorious for the lack of TPO activity. Thus, future studies using a thyrocyte model expressing a functional TPO protein are necessary to clarify the functional significance of the herein described novel regulatory pathway.

## Conclusion

The present results show that the rat TPO gene is a SREBP target gene and that SREBP-dependent transactivation is mediated by an approximately 80 bp region within the first intron of the TPO gene which contains two isolated and two overlapping SREBP-binding elements. In connection with our recent finding that the rat NIS gene, which like the TPO gene is essential for TH synthesis, is also a SREBP target gene in the thyroid, the present findings suggest that SREBPs may be novel targets for pharmacological modulation of TH synthesis. We have shown recently that the main hormonal regulator of the thyroid, TSH, stimulates SREBP expression, which provides a plausible explanation for the recent observation that TSH stimulates expression of genes responsible for fatty acid and cholesterol synthesis in thyrocytes [12]–[14]. The physiological meaning of this mechanism likely is to provide membrane lipids for growth and proliferation of thyrocytes, which is stimulated by TSH. The physiological significance of the present observation that SREBPs also mediate regulation of genes involved in TH synthesis (TPO, NIS [10]) may be interpreted as a mechanism to coordinate lipid and TH synthesis in growing and proliferating thyrocytes. This indicates that SREBPs, at least in the thyroid, are more than just master regulators of lipid synthesis [35], [36]. To provide convincing evidence that this novel regulatory pathway is also of relevance *in vivo* for TH synthesis deserves additional experiments using a reconstituted or follicular model, which facilitates studying the functional consequence of the herein described regulatory pathway with respect to iodine oxidation, thyroglobulin incorporation, and TH synthesis.

## References

[1] Dai G, Levy O, Carrasco N (1996) Cloning and characterization of the thyroid iodide transporter. Nature 379: 458–460. 8559252 10.1038/379458a0

[2] Ruf J, Carayon P (2006) Structural and functional aspects of thyroid peroxidase. Arch Biochem Biophys 445: 269–277. 16098474 10.1016/j.abb.2005.06.023

[3] Colin IM, Denef JF, Lengelé B, Many MC, Gérard AC (2002) Recent insights into the cell biology of thyroid angiofollicular units. Endocr Rev 34: 209–238. 10.1210/er.2012-1015 PMC3610675 23349248

[4] Gérard AC, Many MC, Daumerie C, Costagliola S, Miot F, et al (2002) Structural changes in the angiofollicular units between active and hypofunctioning follicles align with differences in the epithelial expression of newly discovered proteins involved in iodine transport and organification. J Clin Endocrinol Metab 87: 1291–1299. 11889201 10.1210/jcem.87.3.8278

[5] Vassart G, Dumont JE (1992) The thyrotropin receptor and the regulation of thyrocyte function and growth. Endocr Rev 13: 596–611. 1425489 10.1210/edrv-13-3-596

[6] Laglia G, Zeiger MA, Leipricht A, Caturegli P, Levine MA, et al (1996) Increased cyclic adenosine 3′,5′-monophosphate inhibits G protein-coupled activation of phospholipase C in rat FRTL-5 thyroid cells. Endocrinology 137: 3170–3176. 8754735 10.1210/endo.137.8.8754735

[7] Levy O, Dai G, Riedel C, Ginter CS, Paul EM, et al (1997) Characterization of the thyroid Na+/I− symporter with an anti-COOH terminus antibody. Proc Natl Acad Sci USA 94: 5568–5573. 9159113 10.1073/pnas.94.11.5568 PMC20819

[8] Nicola JP, Nazar M, Mascanfroni ID, Pellizas C-G, Masini-Repiso AM (2010) NF-κB p65 subunit mediates lipopolysaccharide-induced Na(+)/I(-) symporter gene expression by involving functional interaction with the paired domain transcription factor Pax8. Mol Endocrinol 24: 1846–1862. 20667985 10.1210/me.2010-0102 PMC5417406

[9] Nazar M, Nicola JP, Vélez ML, Pellizas CG, Masini-Repiso AM (2012) Thyroid peroxidase gene expression is induced by lipopolysaccharide involving nuclear factor (NF)-κB p65 subunit phosphorylation. Endocrinology 153: 6114–6125. 23064013 10.1210/en.2012-1567

[10] Ringseis R, Rauer C, Rothe S, Gessner DK, Schütz LM, et al (2013) Sterol regulatory element-binding proteins are regulators of the NIS gene in thyroid cells. Mol Endocrinol 27: 781–800. 23542164 10.1210/me.2012-1269 PMC5416757

[11] Horton JD, Goldstein JL, Brown MS (2002) SREBPs: activators of the complete program of cholesterol and fatty acid synthesis in the liver. J Clin Invest 109: 1125–1131. 11994399 10.1172/JCI15593 PMC150968

[12] Grieco D, Beg ZH, Romano A, Bifulco M, Aloj SM (1990) Cell cycle progression and 3-hydroxy-3-methylglutaryl coenzyme A reductase are regulated by thyrotropin in FRTL-5 rat thyroid cells. J Biol Chem 265: 19343–19350. 2229080

[13] Aloj SM, Grieco D, Kohn AD, Nikodem VM, Kohn LD (1990) Thyrotropin regulation of malic enzyme in FRTL-5 rat thyroid cells. Mol Endocrinol 4: 611–622. 2280778 10.1210/mend-4-4-611

[14] Bifulco M, Perillo B, Saji M, Laezza C, Tedesco I, et al (1995) Regulation of 3-hydroxy-3-methylglutaryl coenzyme A reductase gene expression in FRTL-5 cells. I. Identification and characterization of a cyclic AMP-responsive element in the rat reductase promoter. J Biol Chem 270: 15231–15236. 7797507 10.1074/jbc.270.25.15231

[15] Espenshade PJ, Li WP, Yabe D (2002) Sterols block binding of COPII proteins to SCAP, thereby controlling SCAP sorting in ER. Proc Natl Acad Sci U S A 99: 11694–11699. 12193656 10.1073/pnas.182412799 PMC129331

[16] Goldstein JL, Rawson RB, Brown MS (2002) Mutant mammalian cells as tools to delineate the sterol regulatory element-binding protein pathway for feedback regulation of lipid synthesis. Arch Biochem Biophys 397: 139–148. 11795864 10.1006/abbi.2001.2615

[17] Cartharius K, Frech K, Grote K, Klocke B, Haltmeier M, et al (2005) MatInspector and beyond: promoter analysis based on transcription factor binding sites. Bioinformatics 21: 2933–2942. 15860560 10.1093/bioinformatics/bti473

[18] Inoue S, Yoshinari K, Sugawara M, Yamazoe Y (2011) Activated sterol regulatory element-binding protein-2 suppresses hepatocyte nuclear factor-4-mediated Cyp3a11 expression in mouse liver. Mol Pharmacol 79: 148–156. 20926756 10.1124/mol.110.068577

[19] Wen G, Ringseis R, Eder K (2010) Mouse OCTN2 is directly regulated by peroxisome proliferator-activated receptor α (PPARα) via a PPRE located in the first intron. Biochem Pharmacol 79: 768–776. 19819229 10.1016/j.bcp.2009.10.002

[20] Nohturfft A, Yabe D, Goldstein JL, Brown MS, Espenshade PJ (2000) Regulated step in cholesterol feedback localized to budding of SCAP from ER membranes. Cell 102: 315–323. 10975522 10.1016/s0092-8674(00)00037-4

[21] Sheng Z, Otani H, Brown MS, Goldstein JL (1995) Independent regulation of sterol regulatory element-binding proteins 1 and 2 in hamster liver. Proc Natl Acad Sci U S A 92: 935–938. 7862668 10.1073/pnas.92.4.935 PMC42611

[22] DeBose-Boyd RA, Ou J, Goldstein JL, Brown MS (2001) Expression of sterol regulatory element-binding protein 1c (SREBP-1c) mRNA in rat hepatoma cells requires endogenous LXR ligands. Proc Natl Acad Sci U S A 98: 1477–1482. 11171976 10.1073/pnas.98.4.1477 PMC29282

[23] Repa JJ, Liang G, Ou J, Bashmakov Y, Lobaccaro JM, et al (2000) Regulation of mouse sterol regulatory element-binding protein-1c gene (SREBP-1c) by oxysterol receptors, LXRα and LXRβ. Genes Dev 14: 2819–2830. 11090130 10.1101/gad.844900 PMC317055

[24] Sato R (2010) Sterol metabolism and SREBP activation. Arch Biochem Biophys 501: 177–181. 20541520 10.1016/j.abb.2010.06.004

[25] Shimomura I, Shimano H, Horton JD, Goldstein JL, Brown MS (1997) Differential expression of exons 1a and 1c in mRNAs for sterol regulatory element binding protein-1 in human and mouse organs and cultured cells. J Clin Invest 99: 838–845. 9062340 10.1172/JCI119247 PMC507890

[26] Amemiya-Kudo M, Shimano H, Hasty AH, Yahagi N, Yoshikawa T, et al (2002) Transcriptional activities of nuclear SREBP-1a, -1c, and -2 to different target promoters of lipogenic and cholesterogenic genes. J Lipid Res 43: 1220–1235. 12177166

[27] Briggs MR, Yokoyama C, Wang X, Brown MS, Goldstein JL (1993) Nuclear protein that binds sterol regulatory element of low density lipoprotein receptor promoter. I. Identification of the protein and delineation of its target nucleotide sequence. J Biol Chem 268: 14490–14496. 8390995

[28] Nara TY, He WS, Tang C, Clarke SD, Nakamura MT (2002) The E-box like sterol regulatory element mediates the suppression of human Delta-6 desaturase gene by highly unsaturated fatty acids. Biochem Biophys Res Commun 296: 111–117. 12147235 10.1016/s0006-291x(02)00851-3

[29] Amemiya-Kudo M, Shimano H, Yoshikawa T, Yahagi N, Hasty AH, et al (2000) Promoter analysis of the mouse sterol regulatory element-binding protein-1c gene. J Biol Chem 275: 31078–31085. 10918064 10.1074/jbc.M005353200

[30] Rozman D, Fink M, Fimia GM, Sassone-Corsi P, Waterman MR, et al (1999) Cyclic adenosine 3′,5′-monophosphate(cAMP)/cAMP-responsive element modulator (CREM)-dependent regulation of cholesterogenic lanosterol 14alpha-demethylase (CYP51) in spermatids. Mol Endocrinol 13: 1951–1962. 10551787 10.1210/mend.13.11.0377

[31] Dubchak I, Brudno M, Loots GG, Pachter L, Mayor C, et al (2000) Active conservation of noncoding sequences revealed by three-way species comparisons. Genome Res 10: 1304–1306. 10984448 10.1101/gr.142200 PMC310906

[32] Ericsson J, Jackson SM, Kim JB, Spiegelman BM, Edwards PA (1997) Identification of glycerol-3-phosphate acyltransferase as an adipocyte determination and differentiation factor 1- and sterol regulatory element-binding protein-responsive gene. J Biol Chem 272: 7298–7305. 9054427 10.1074/jbc.272.11.7298

[33] Guan G, Dai PH, Osborne TF, Kim JB, Shechter I (1997) Multiple sequence elements are involved in the transcriptional regulation of the human squalene synthase gene. J Biol Chem 272: 10295–10302. 9092581 10.1074/jbc.272.15.10295

[34] Tabor DE, Kim JB, Spiegelman BM, Edwards PA (1999) Identification of conserved cis-elements and transcription factors required for sterol-regulated transcription of stearoyl-CoA desaturase 1 and 2. J Biol Chem 274: 20603–20610. 10400691 10.1074/jbc.274.29.20603

[35] Yokoyama C, Wang X, Briggs MR, Admon A, Wu J, et al (1993) SREBP-1, a basic-helix-loop-helix-leucine zipper protein that controls transcription of the low density lipoprotein receptor gene. Cell 75: 187–197. 8402897

[36] Hua X, Yokoyama C, Wu J, Briggs MR, Brown MS, et al (1993) SREBP-2, a second basic-helix-loop-helix-leucine zipper protein that stimulates transcription by binding to a sterol regulatory element. Proc Natl Acad Sci USA 90: 11603–11607. 7903453 10.1073/pnas.90.24.11603 PMC48032

